# Confocal measurement of microplastics uptake by plants

**DOI:** 10.1016/j.mex.2019.11.023

**Published:** 2019-12-03

**Authors:** Lianzhen Li, Yongming Luo, Willie J.G.M. Peijnenburg, Ruijie Li, Jie Yang, Qian Zhou

**Affiliations:** a Key Laboratory of Coastal Zone Environmental Processes and Ecological Remediation, Yantai Institute of Coastal Zone Research (YIC), Chinese Academy of Sciences (CAS), China; b Key Laboratory of Soil Environment and Pollution Remediation, Institute of Soil Science (ISSAS), Chinese Academy of Sciences (CAS), China; c National Institute of Public Health and the Environment, Center for Safety of Substances and Products, P.O. Box 1, 3720 BA, Bilthoven, the Netherlands; d Institute of Environmental Sciences (CML), Leiden University, Leiden, the Netherlands

**Keywords:** A simple and rapid approach for imaging of microplastics in plant, Microplastics, Confocal laser scanning microscopy, Plant uptake, Fluorescent imaging

## Abstract

Microplastics (MPs, plastics 100 nm–5 mm in diameter) are estimated to accumulate in agricultural soils in quantities that exceed the total MP burden in ocean waters. Despite a wealth of information relating to the accumulation of MPs in aquatic species, there is little information on the uptake of MPs by terrestrial plants. Information about location of MPs in plant tissues is critical to understand the modes of their interaction with plants. Polystyrene (PS) is one of the most commonly used plastic polymers worldwide and it is often found in MPs sampled in the environment. The performance of traditional detection methods (i.e., transmission electron microscopy, TEM and scanning electron microscopy, SEM) for nanoparticles is limited due to the extensive sample preparation and the limited field of view. Here we report an approach for the imaging of different sizes of PS plastic beads (ranging from submicrometer to micrometer-sized) within plant tissues by using confocal laser scanning microscope (CLSM). Fluorescent dye Nile blue or 4-chloro-7-nitro-1,2,3-benzoxadiazole were encapsulated into the PS microbeads through swelling method and they were used to detect the localization of PS beads in the root and the green tissue respectively.

• This is a simple and rapid approach for imaging of MPs in plant.
• The fluorescent dyes can produce bright and stable emission signals that are distinguishable from the autofluorescence background of plant tissues.
• The dyes leakage in the aqueous phase can be assumed to be negligible.

## Graphical abstract

**Figure:**
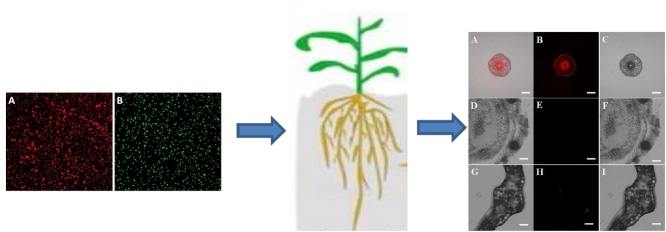

**Specification Table**

**Table:** 

Subject Area:	Environmental Science
More specific subject area:	*Environmental Impact/Environmental Ecotoxicology*
Method name:	A simple and rapid approach for imaging of microplastics in plant
Name and reference of original method:	*NO reference*
Resource availability:	*No resource available*

## Method details

### Synthesis and characterization of fluorescently labeled polystyrene particles

Fluorescent PS particles (without functionalization) were customized synthesized from Da'e Scientific Co., Ltd. (Tianjin, China). The two sizes of PS microbeads were first synthesized by means of dispersion polymerization and a mini-emulsion polymerization process [1]. Then, the fluorescent dye Nile blue (NB) or 4-chloro-7-nitro-1,2,3-benzoxadiazole (NBD-Cl) was immobilized into the polymer matrix through a swelling method by dispersing PS beads in 0.25 % sodium dodecyl sulfate (SDS) and taking methylene dichloride as swelling agent [2]. The labeled beads were centrifuged and washed with ethanol and deionized water to remove any free dyes until there is no fluorescence detected in the supernatant. The stock solutions were supplied as 10 mg mL^−1^ suspensions in ultrapure water (18.2 MΩ, Millipore, USA). The PS beads had primary nominal sizes of ∼0.2 μm and ∼2.0 μm. The actual sizes of the 0.2 μm and 2.0 μm PS microbeads were determined to be 0.23 ± 0.04 μm and 1.98 ± 0.08 μm using SEM (Fig. S1).

These two fluorescent dyes were used to detect the presence of PS beads because the red fluorescence from NB is indistinguishable from the red autofluorescence emitted by chloroplasts and the green fluorescence from NBD-Cl is indistinguishable from the green autofluorescence in root tissues (Fig. S2). Thus, in this study PS-NB was used to detect PS beads in roots, and PS-NBD-Cl was used to detect PS beads in stems and leaves. To assess the stability of the fluorescence, we analyzed the loss of fluorescence intensity after exposure of the PS beads in Hoagland solutions within plants for 3, 6, 12, 24, 48, and 72 h using a Synergy H1 microplate reader (Bio Tek, Winooski, VT, USA) with excitation and emission wavelengths of 488 nm and 518 nm or 635 nm and 680 nm. The leakage of fluorescence was assessed by measuring the fluorescence before and after filtration through a 0.1-μm syringe filter at the end of the indicated incubation periods. For Nile Blue labeled 0.2 μm and 2.0 μm PS beads suspensions, the amount of dye after filtration was below to the microplate reader detection limit. The dissolved dye 4-chloro-7-nitro-1, 2, 3-benzoxadiazole release into the solution did not exceed 3.7 % for the PS beads (Fig. S3). Consequently, the presence of fluorescent markers in the aqueous phase can be assumed to be quite negligible.

The average hydrodynamic diameter and the zeta potential of the PS beads in Hoagland solution were determined by dynamic laser scattering (DLS) using a Malvern Zetasizer Nano-ZS90 (ZEN3590, UK). Both stock suspensions were sonicated for 2 min using a sonicator (Branson Ultrasonic, Danbury, CT, USA) and were then diluted to obtain the final concentrations of the beads used in the tests. The average PS beads hydrodynamic diameter was 0.27 ± 0.04 μm and 2.5 ± 0.3 μm for the 0.2-μm and 2.0-μm PS beads, respectively. The surface of the MPs was negatively charged, with the 0.2-μm beads having a more negative zeta potential (−42.7 ± 0.9 mV) than the 2.0-μm beads (−12.1 ± 0.6 mV).

#### Plant materials and growth conditions

Wheat was chosen as a model plant in this study because of its wide production as one of the main world-wide foodstuffs in the world. Seeds of wheat (*Triticum aestivum* L., Zhongmai 9) were supplied by the Chinese Academy of Agricultural Sciences, Beijing, China. Wheat seeds of an almost uniform size were selected and surface sterilized by treatment with a 10 % NaClO solution for 5 min. Subsequently, the seeds were washed three times with deionized water to remove the residual NaClO solution and were then transferred to moistened filter paper and incubated in the dark at 25 °C to induce germination. After four days, four seedlings with uniform size were transferred to a 1-L beaker containing Hoagland solution. The beaker was covered with a cap and placed in a growth chamber (illumination of 250 μmol m^−2^ s^−1^ for a 12 h/12 h day/night photoperiod; 70–80 % relative humidity; 25 ± 2 °C). Each box was equipped with an aquarium aerator to provide oxygen to the roots and to maintain the PS beads in suspension. The wheat plants were exposed to PS microbeads for 10 days. The experiment was repeated three times.

#### Imaging by confocal laser scanning microscopy

Following exposure to PS beads, the roots were removed and washed thoroughly with distilled water. Subsequently, fresh root (mature zone) and stem (2 cm from the base of the stem) segments were collected and embedded in 4 % agarose. The root and stem samples and the leaf blades (with the primary vein) were sectioned into 40- and 100-μm thick sections, respectively, using a vibrating microtome (VT1200S Vibrotome, Leica, Vienna). Semi-thin sections of the samples were placed on a glass slide covered with a coverslip, and a few drops of PBS were added to keep the sample hydrated. The sectioned tissue was inspected using a confocal laser scanning microscope (FluoView FV1000; Olympus, Japan) with excitation/emission wavelengths of 488/515 nm and 635/680 nm for NBD-Cl and NB, respectively. Transverse and longitudinal sections of at least three plants were examined from each treatment group before representative images were selected. Images were captured with a SPOT camera and imported into imaging software Fluoview FV 10 for the compound microscope. Pictures have brightness and contrast enhancement.

#### Observation by scanning electron microscopy

Selected samples from the roots, stems and fully expanded leaves near the primary veins were excised, sectioned into small pieces and frozen in liquid nitrogen. The samples were then freeze-dried and coated with gold for 60 s (ca. 1 nm thickness of gold) by a Sputter Coater (Cressington model 108, Ted Pella Inc., U.S.), and then detected by a scanning electron microscope (SEM; SU8010, Hitachi, Japan). The cross sections were viewed at an accelerating potential of 20 kV under high vacuum mode with backscatter detection. Images were captured at different magnifications. For each species, at least three plants were examined for each treatment group. Digital photographs were taken using an EVO 40 scanning electron microscope (Zeiss).

#### Methods validation

**Fig. 1:**
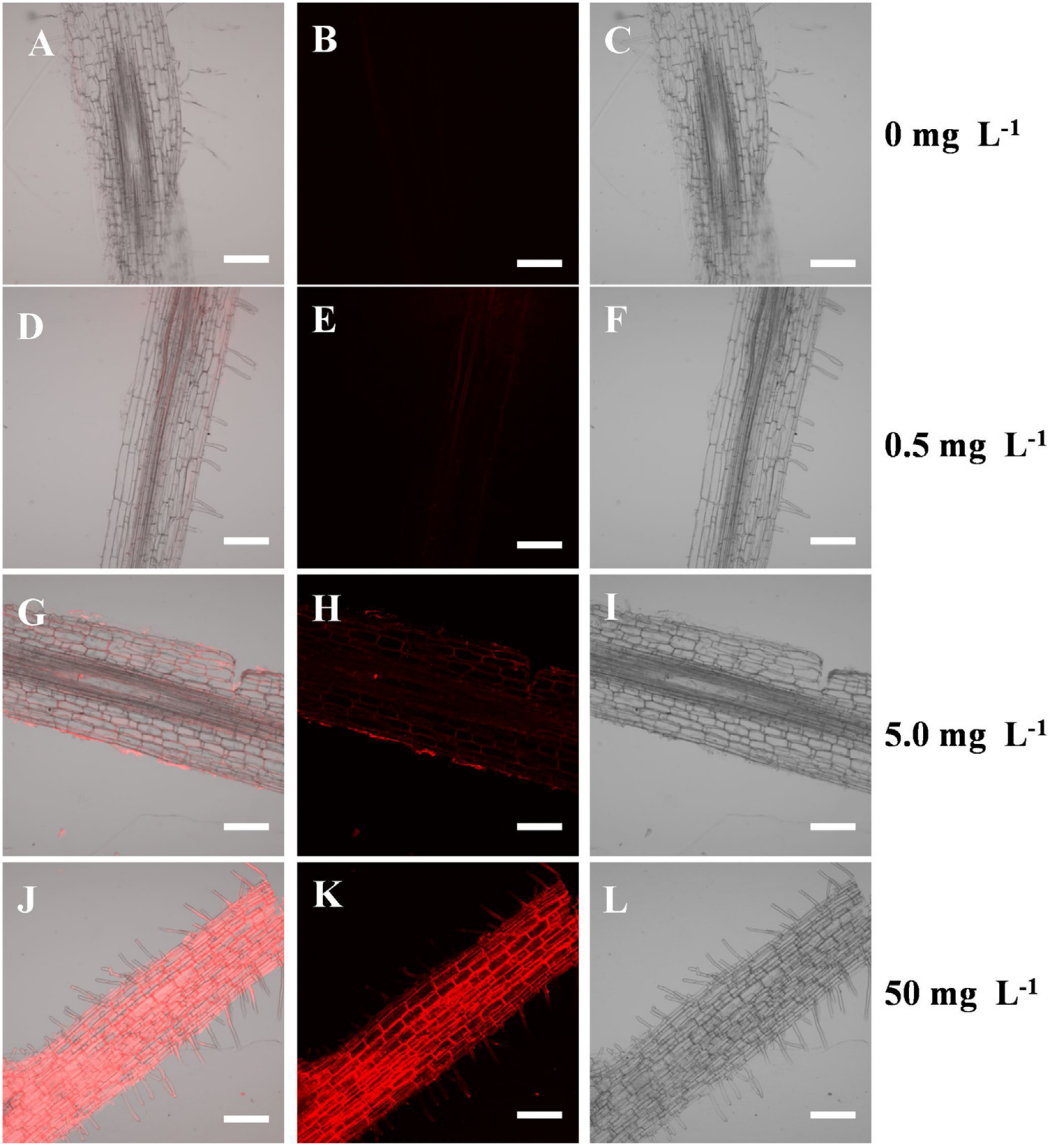
Longitudinal sections of the mature zone of roots of wheat grown in Hoagland solution with 0.2 μm PS microbeads at concentrations of 0 (A–C), 0.5 (D–F), 5.0 (G–I) or 50 (J–L) mg L^−1^ for 10 d. PS beads were labeled with Nile blue. The accumulation of PS beads was analyzed under bright-field conditions and in the red channel using CLSM. Bar = 100 μm.

**Fig. 2:**
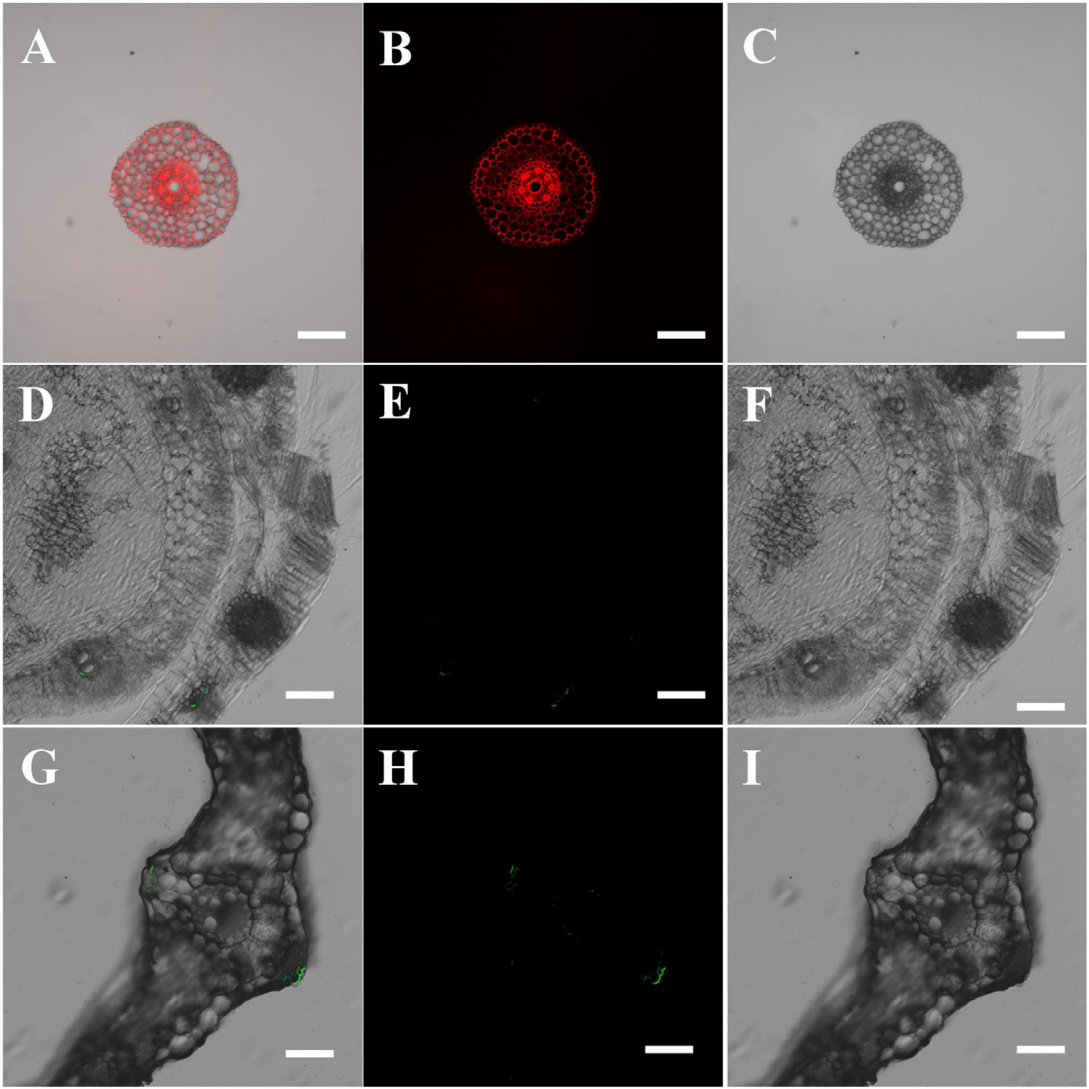
Confocal images of cross sections of a wheat root (A–C), stem (D–F) and leaf (G–I) treated for 10 d with a 50 mg L^−1^ solution of 0.2 μm fluorescently labelled polystyrene (PS) microbeads. Images A, D, and G are the corresponding merged images of image B and C, E and F, H and I.

**Fig. 3:**
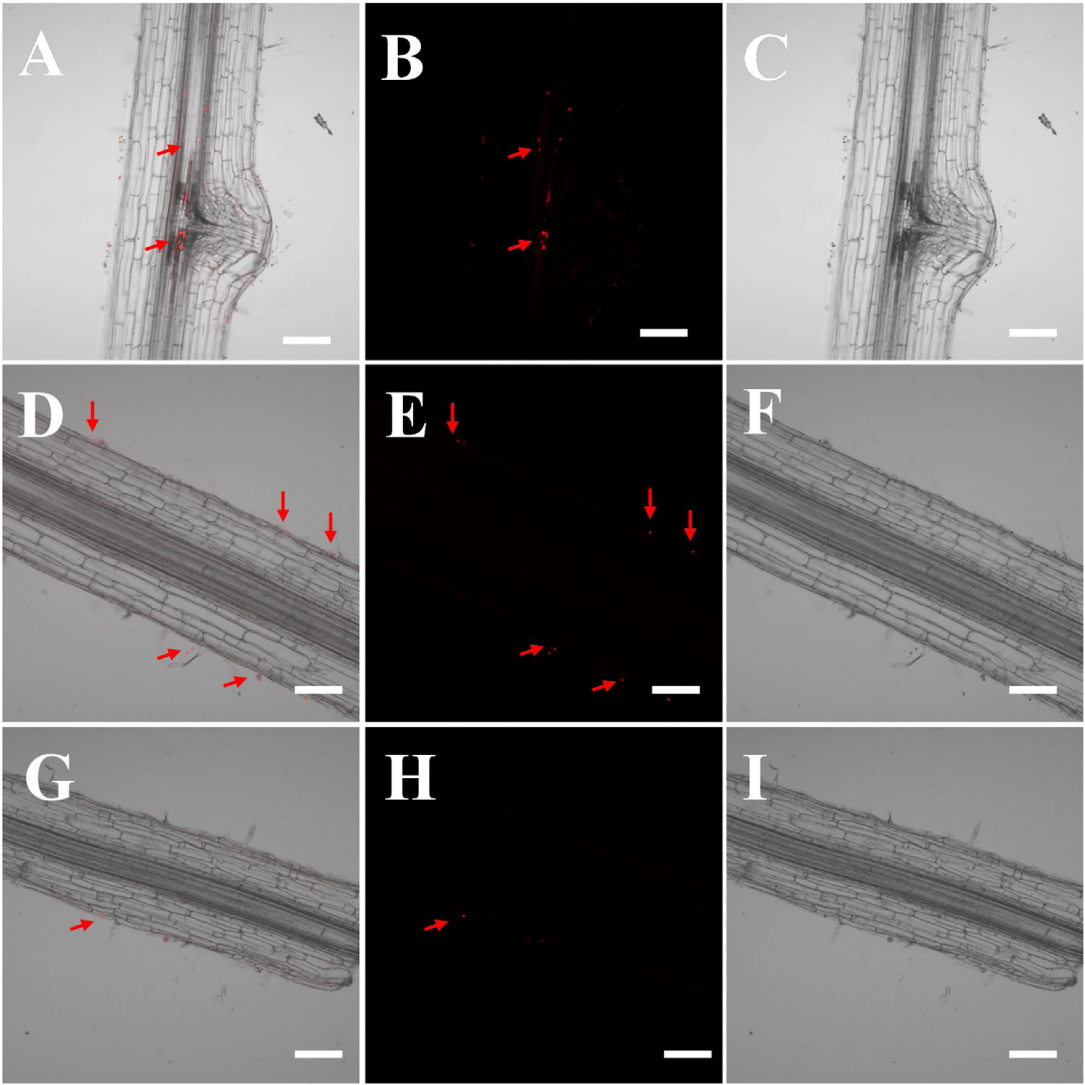
Confocal images of longitudinal sections in the root zone 70 mm from the root apex of wheat treated for 10 d with a 50 mg L^−1^ solution of 2 μm (A–C), 5 μm (D–F) or 7 μm (G–I) polystyrene (PS) microbeads labeled with Nile blue. The accumulation of PS beads was analyzed in the red channel using CLSM. These are merged bright-field and confocal images. Bar = 100 μm.

We used two fluorescent markers (Nile blue and 4-chloro-7-nitro-1, 2, 3-benzoxadiazole, which emitted a red and green fluorescence signal, respectively, Fig. S4) to track PS beads in plant tissues and found fluorescence to be a sensitive and reliable detection method. Plants without exposure of fluorescent PS beads were used as a control to adjust the detector gain and establish the baseline. All images were acquired with the same detector gain to ensure comparable relative intensities. Under the imaging conditions used, sections from untreated control plants showed no detectable autofluorescence at specific excitation (Fig. S5). When roots were treated with fluorescent PS microbeads, the beads could be identified by their light fluorescence. The roots of wheat grown in 0.2 μm microbead suspension showed clear concentration-dependent fluorescence in a wide range from 0.5–50 mg L^–1^ (Fig. 1), thus providing evidence for increased accumulation of beads by wheat plants at higher treatment concentrations. Based on the strong fluorescence signal obtained in the roots of wheat when grown in the 50.0 mg L^–1^ suspension of 0.2 μm PS beads, 50.0 mg L^–1^ suspensions of different sizes of microbeads (0.2. 2.0, 5.0. or 7.0 μm) were used for investigations in the hydroponic exposure medium. Significant fluorescence was observed in the roots, shoots and leaves of wheat exposed to 0.2 μm beads (Fig. 2). Very few luminescence signals were observed in the vascular system or in the epidermis of wheat roots for 2.0 μm and almost none for 5.0 μm and 7.0 μm beads (Fig. 3). The presence of aggregates of the 0.2 μm PS beads, mostly in the xylem and on the cell walls of the cortex tissue in the wheat root, indicated that the beads passed through the intercellular space via the apoplastic transport system (Fig. 2). Once inside the central cylinder, particles can move toward the aerial parts of a plant though the xylem, following the transpiration stream. The 0.2 μm PS beads were transferred from the roots to the stems and leaves via the vascular system through the apoplastic pathway to the leaf vein vasculature (Fig. 2). No fluorescence was observed in the stem and leaves for 2.0 μm PS beads.

Briefly, our observations demonstrated the usefulness of using fluorescent labeled MPs for studying their localization within the whole plant tissues. The results presented here clearly demonstrate the uptake and transport of PS microbeads by plant roots and the *in vivo* distribution into the stem and leaves. Our work reported a visualization method to detect the accumulation and distribution of MPs in plant tissues, which could be beneficial to profoundly understand the biological effects and the modes of interaction of MPs with plants.

## Additional information

Microplastics may present an attributable risk to ecosystem and human health, and their presence in the biosphere has become a global concern. Terrestrial edible plants are for instance directly exposed to MPs when organic manure, sewage sludge as fertilizer, or plastic mulching [[3], [4], [5]] are applied to agricultural soil. Despite a wealth of information on the accumulation of MPs in aquatic species [[6], [7], [8]], very limited information on the uptake and accumulation of MPs by higher plants exists to date. With the increasing amounts of smaller plastic particles directly emitted into the environment as well as secondary particles formed by degradation of plastics, it is critical to understand the interactions between MPs and plants. Studies have shown the possibility of plants accumulating small size microplastics [9] and being affected either positively or negatively depending on plant type [[10], [11], [12], [13], [14]]. Information regarding the accumulation of MP by plants is still very limited and the underlying mechanism of uptake is currently not clear. Therefore, more studies are required for better understanding the accumulation and distribution of MPs in the plant. For intracellular imaging of MPs, scanning electron microscopy (SEM) provides high resolution, showing MP shapes and sizes in addition to their location [9]. However, identifying MPs using SEM or TEM requires high magnification, resulting in a limited field of view. In addition, the extensive sample preparation and fixation required for SEM or TEM makes it challenging to explore intracellular trafficking of MPs in real time. Visualization of fluorescent MPs is a simple, rapid, and non-invasive approach for the imaging of MPs within plant tissues. It is capable of rapidly screening whole tissues by using confocal laser scanning microscope (CLSM) and sample preparations are simple. No additional optical or imaging system is required for this approach.

## Declaration of Competing Interest

The authors report no conflicts of interest. The authors alone are responsible for the content and writing of the paper.
