# Supplementary material for: Confocal measurement of microplastics uptake by plants
Source: MethodsX. 2019 Dec 3;7:100750. doi: 10.1016/j.mex.2019.11.023 (PMC6993004; doi:10.1016/j.mex.2019.11.023)
Supplement: Supplementary file 1 [file mmc1.docx]

**Fig. S1.** Representative SEM images of 0.2 (A) and 2 um (B) PS microbeads.

**Fig. S2** Confocal images of cross (A, B, C) section of wheat root, stem (G, H, I) and leaf (J, K, L) with various excitation wavelengths.

**Fig. S3** Leakage of the fluorescent dye 4-chloro-7-nitro-1,2,3-benzoxadiazole, as assessed after 3, 6, 12, 24, 48 and 72 hours of exposure under the same conditions as used in the uptake experiments. The free fluorescence of the PS particles is shown as a percentage of the total fluorescence intensity ± SD, n=4.

**Fig. S4** Confocal images of 0.2 μm red (A) and green (B) fluorescently labelled polystyrene (PS) microbeads.

**Fig.S5** Confocal images of a cross section of a wheat root treated for 10 d without fluorescently labelled polystyrene (PS) microbeads. Images A, D, G and J are the corresponding merged images of image B and C, E and F, H and I.
